# Reviews and Book Notices

**Published:** 1878-02

**Authors:** 


### Reviews and Book Notices.

---

HOSPITALS: THEIR HISTORY, ORGANIZATION AND CONSTRUCTION. By W. Gill Wylie, M. D. New York: Appleton & Co., 1877.

In a preface of two pages, the author discloses the source of the inspiration which prompted him to write the Boylston prize essay for 1876. During his residence as a medical officer in Bellevue Hospital, he had ample " opportunity for seeing the bad effects of poor nursing and defective construction on the welfare of patients." "The sanitary condition of the hospital was shocking," etc. His subsequent acquaintance with less ancient and more pretentious buildings (e. g., the elegant New York Hospital, the name of which seems to enrage Dr. Wylie, as the classical red flag infuriates the pugnacious bovine), has not materially changed his earlier impressions ; hence the present essay.

The first chapter relates to the " history of the origin and development of hospitals," a somewhat rambling and incoherent mixture of history and tradition, culminating in the conclusion that " the credit of the origin of the first hospitals is not due to medicine, but to religion."

In the second chapter, entitled "the relations of hospitals to pauperism," are tersely stated some forcible, though by no means original, objections to charity hospitals as they are. That they tend to foster idleness, helplessness, and their natural results, pauperism and crime; that the unrestricted offer of gratuitous relief removes the stimulus of necessity which prompts the average mortal to provide against sickness, is well known to all connected with large hospitals or dispensaries. In support of this position, our author cites the official reports of the New York charities for 1875. In a city of 1,000,000 inhabitants, $10,000,000 were spent in charitable reliefs; 6,000 hospital beds were accessible to all; over 300,000 persons (?) received gratuitous medical advice and medicine. "The truth is, the majority of our hospitals, as they are at present managed, are liable to do more harm than good."

Our author suggests that it would be far more creditable and humane if the money which is so lavishly expended in relieving the sufferings of the poor, were devoted, in part at least, to the removal of the cause of those sufferings. Since many, probably a majority, of the diseases treated in hospitals can be traced to the ignorant disregard of sanitary laws manifested in the homes and habits of the poor, sanitary education could and should be substituted,in part at least, for indiscriminate alms-giving. Dr. Wylie thinks, further, that on sanitary grounds alone, most of our existing hospitals are inferior, as sick rooms, to the homes of the poor, however humble the latter may be.

The third chapter—"organization and management"—is prefaced by the remark that "more than four-fifths of all that we have been able to find written on hospitals is mainly about their construction. Many times the results of carelessness on the part of the doctors, unwise and fraudulent management, poor, untrained nurses, bad food, and general uncleanliness, have been unjustly attributed to faults in the plan of construction." He hints that results would be more satisfactory if science had a larger, politics and social station a smaller, representation on boards of hospital managers; if superintendents were invariably medical men (the skeptic is respectfully referred to our Cook County Hospital); if ladies were associated with gentlemen in the general inspection of the hospital work; and if training-schools for nurses were more generally established.

Five chapters are devoted to the construction of a civil hospital, discussing in detail the character of the buildings, arrangement of the ward and its appurtenances, warming and ventilation, furniture, isolation wards, mortuary, drug-room, etc. The author considers it proven that the one-story pavilion plan is least objectionable; that the wards devoted to contagious and infectious diseases should be destroyed at short intervals; that wards should contain not more than twenty-five beds each; that at least 1,800 cubic feet of space, with an area of 124 square feet, should be allowed each patient.

Another chapter presents some Utopian ideas on the relation of the medical school to the hospital. The last section discusses "hospital buildings now in use," containing plans and descriptions of the most notable examples in Europe and America.

As a literary work, the book is a failure; the style, while sufficiently plain, is not sufficiently terse and accurate for scientific exposition. The evident desire to make a "complete treatise" on the subject, has encumbered the book with considerable useless lumber. Yet there is evidence of fair experience, close observation, ripe scholarship and original thought. The essay is everywhere stamped with the impress of the author's individuality, and though undue prominence is given to certain pet ideas as to the details of construction, the views of others are fairly stated and plainly criticised.

The book may be read with much interest and profit, not only by hospital managers and public sanitarians, but by general practitioners. W. T. B.

Cyclopædia of the Practice oe Medicine. Edited by Dr. H. Von Ziemssen, professor, etc. Vol. XV. Diseases of the Kidney. By Prof. Carl Bartels, of Kiel, and Prof. Wilhelm Ebstein, of Göttingen; translated by Reginald Southey, M. D., Oxon, of London, and Robert Bertolet, M. D., of Philadelphia; Albert D. Buck, M. D., editor of American edition. New York: Wm. Wood & Co.; pp. 796.

This volume opens with biographical sketches of the authors, Carl Bartels, of Kiel, and Wm. Ebstein, of Göttingen. The former treats of the structural diseases of the kidneys and the general symptoms of renal affections, while the latter considers more particularly affections of the renal pelvis, ureter, diseases of the connective tissue of the gland and anomalies in the position, form, and number of the kidneys.

Prof. Bartels discusses the subject of albuminuria very thoroughly, and says that even at the present time the different views held by Bright, Prout, Graves, and others, are not reconciled, and while some physicians consider the term albuminuria *as an equivalent for kidney* disease, there are others who attribute all such cases to an altered condition of the blood.

He divides the cases of albuminuria into two classes: first, those in which it is merely the accompaniment of other acute disease, dependent upon secondary disturbance of the circulation through the kidneys; and, second, that class of cases where albumen in the urine is the prominent symptom, due to the fact that the kidneys have suffered an alteration in structure. Passing to the consideration of the consequences of an imperfect depuration of the blood in renal disease, he discusses the different interpretations which have been given to the series of symptoms to which the term uræmia is applied. He attributes our inability to discover the essential nature of uræmia to the circumstance that our attention has been chiefly devoted to experiments upon animals instead of to clinical observations. After disproving the theory that the symptoms of uræmia are due to carbonate of ammonia in the blood, and showing that the accumulation of urea is not a necessary factor, he comes to the conclusion "that the symptoms are all caused by some disorder of the urinary secretion, and that the title of uræmia is rightly attached to them."

He calls attention to the marked tendency to inflammatory affections exhibited by patients who have kidney disease, and says that these inflammatory complications occurring in other organs, are the cause of death more frequently than dropsy, uræmia and apoplexy reckoned together.

Under the term, the diffuse diseases of the kidneys, he devotes nearly four hundred pages to the consideration of those diseases, which have been grouped together under the title of Bright's disease. Prefacing this with an interesting historical sketch, he says that "his clinical experience and pathologico-anatomical investigations," have led him to distinguish the following distinct processes:

1st. Active and passive hyperæmia.

2d. The acute and chronic parenchymatous inflammation of the kidneys.

3d. The renal affections of cholera.

4th. Intestinal inflammation producing the contracted kidney.

5th. Amyloid degeneration of the kidneys.

He divides the causes of acute nephritis into two categories: the first embraces all those causes where certain specific noxious substances are carried by the blood to the kidney; and the second, those causes which act upon the vessels of the kidneys, and upon the circulation of the blood through them so as to favor inflammatory changes. Under the head of acute nephritis, he considers the nephritis of pregnancy, but, after refuting all the explanations formerly advanced as to its mode of origin, confesses his inability to furnish an explanation of his own.

Next to chronic suppuration, he mentions marsh miasm as a decidedly frequent cause of chronic parenchymatous nephritis, while, in opposition to the opinions of a great many physicians, he does not think the long-continued use of mercury in syphilis, or that alcoholic excesses can be charged with being the cause of it; and in his chapter on renal cirrhosis he is equally emphatic in his protest against the view that the abuse of spirituous liquors favors the development of the genuine contracted kidney. He expresses strongly his belief that genuine renal contraction, the third stage of Bright's disease, is not preceded by an inflammatory swelling of the organ.

Prof. Bartels concludes his part of this volume with an account of amyloid degeneration of the kidney, which he considers the local manifestation of a general constitutional disease.

Prof. Ebstein, in the first part of his section, considers the traumactic, idiopathic, pyæmic and metastic forms of renal inflammations which lead to the formation of abscesses. He then describes pyelitis and pyo-nephritis in the same chapter, as the extension of the inflammation from the pelvis to the renal parenchyma very frequently occurs.

He follows with chapters on tumors of the kidney, foreign bodies in the kidney, animal parasites of the kidney, and, lastly, anomalies in position of the kidney and diseases of the renal vessels.

In referring to the movable kidney, he says that the statement made by Rayer that the female sex is particularly liable to this anomaly has been completely verified. Prof. Ebstein attributes it to the over distention of the abdominal walls, which occurs after frequent pregnancies, rather than to tight lacing.

This volume contains a vast amount of information, and is equal in excellence to any of those preceding it. H. H.

CYCLOPÆDIA OF THE PRACTICE OF MEDICINE. Edited by H. von Ziemssen, Professor of Clinical Medicine in Munich, Bavaria. Vol. XVI. Diseases of the Locomotive Apparatus and General Anomalies of Nutrition, by Prof. H. Senator, of Berlin; Prof. E. Seitz, of Giessen; Poof. H. Immerman, of Basel; and Dr. Birch-Hirschfeld, of Dresden. Translated by E. Buchanan Baxter, M. D., Lond., of London; John Todhunter, M. D., of Dublin; Godfrey Aigner, M. D., and Frank P. Foster, M. D., of New York; and Henry P. Bowditch, M. D., of Boston. Albert H. Buck, M. D., New York, Editor of American Edition. New York: Wm. Wood & Co. 1877.

This translation of the great German work is approaching its termination by degrees. Only five more volumes yet await publication.

The volume just issued is devoted to the non-surgical diseases of the locomotive apparatus, and to general anomalies of nutrition.

The article on rheumatism, written by Senator, is, perhaps, one of the least satisfactory in the whole Cyclopædia. The author uses the term rheumatism as a vague word—a kind of provisional pigeon-hole to contain a great variety of diseases for the present, because we do not at present understand their nature, and cannot, therefore, classify them scientifically. He looks upon it as a temporary word, to be dropped when the advance of pathology shall clear up the obscurity; and he scarcely recognizes such a thing as rheumatic diathesis. Now it is doubtless true that our means of diagnosis are imperfect, and that there are many cases called rheumatic which are not really so, but the man who, on that account, ignores the rheumatic diathesis and its tremendous influence on various forms of disease, is unfit to write the article on rheumatism in a cyclopædia. As might be expected, the discussion of the treatment is hesitating and dubious. There is one very valuable trait, however, exhibited by the author. It is this:

Where he finds that science has failed to enlighten us on any point, he makes a bold statement of what we do not know, instead of resorting to vague and pompous phraseology to bridge over the chasms of our ignorance. He would do science a service if he could induce most of our standard authors to imitate his humility.

The article makes no mention of the new salicylic and salicin treatment, though this is explained by a note of the translator, which states that the discoveries were made after the article was written. The note gives a brief statement of what is known on the subject. The conclusions are that the salicylic acid and the the sodium salicylate have essentially similar effects in reducing the temperature, and rapidly curing acute rheumatism. The sodium salicylate is preferred, and is given in doses of ten or twenty grains every hour or two, until relief is obtained, which occurs in from twelve to thirty-six hours. Afterward it is given less frequently for some time to prevent a relapse. A few cases are recorded where the remedy has produced great prostration, and resulted, as is believed, in the death of one patient. Many of the French physicians believe the remedy is dangerous when any disease of the kidney exists.

The salicin is similar in its effects to the sodium salicylate, and has been experimented with thoroughly by Dr. Maclagan.

After a discussion of the various forms of rheumatism and gout, he takes up rickets and malacosteon, and gives them a pretty full study.

Seitz contributes the next article, which is on "Slight Disorders Caused by Catching Cold."

After this we have a full and valuable discussion of the "General Disorders of Nutrition," by Immermann. This article covers nearly five hundred pages, and considers the subjects of Anæmia, Chlorosis and Corpulence,

The next discussion is upon "Scofulosis and Malignant Lymphoma," by Birch-Hirschfeld. In this he brings out the powerful remedial effects of arsenic on the latter kind of tumor. It seems not improbable that observations on growths of this character led Prof. Atlee to the notion that arsenic is a remedy for cancer in general.

The concluding essay is by Senator on "Diabetes Mellitus," and occupies about two hundred pages.

The volume has a copious index, and its typography is of the finest description. E. A.

A TREATISE ON GONORRHŒA AND SYPHILIS: By Silas Durkee, M. D., etc. Sixth edition, with eight colored illustrations. Philadelphia: Lindsay & Blakiston, 1877; pp. 467.

The sixth edition of this rather pretentious volume is very like its predecessors from the same type. Bearing such resemblance to each other, they recall the individual peculiarities of a character not unfrequently encountered in the world of society.

It is that of the little old-young man. Everybody has seen him. Everyone has observed his artistically fashioned, jaunty clothing, intended to produce the impression that he is still in the hey-day of youth. His sparse hair is so arranged over his scalp as to conceal the ravages made there by the deft fingers of time. His facial wrinkles are offset by the sprightliness of his manner. Yet the leanness of the calf of his leg and the twinges in the muscles of his back occasionally betray the fact that the famous people with whom he is proud to claim acquaintance belonged to a generation that is past.

The dress of the book before us is unexceptionable. In the work of the typographer, the proof-reader and the bookbinder no fault can be found. Even the style of the author, though occasionally careless, is fairly in keeping with the exterior. He who turns, however, to the subject-matter will be disappointed by discovering that the book is really an old one. Its discussions are of questions long settled or dismissed. Its arrangement is defective; its pathology antiquated. We have carefully examined the references in every foot-note, and have not discovered one of a more recent date than 1863. Think of the indefatigable industry of hundreds of syphilographers and authors on the subject of venereal diseases, extending through the last thirteen years, which here bears no fruit! Compare the laborious exposition of each topic in this old-new treatise with the incisive clearness and practical worth of the two other distinctively American works covering the same general ground, and the contrast becomes painful. The author discusses gonorrhœa in women without referring to inflammation of the vulvo-vaginal gland—one of its most important complications. He fails utterly to distinguish between those genital sores which precede syphilis and those which do not. He advises to treat the syphilitic bubo by making a "pretty deep sore" over the enlarged gland with a vesicant, to which afterward mercurial ointment and the muriate of ammonia are applied! Nearly 40 pages are devoted to an attempt to prove that secondary syphilis may not be preceded by primary lesions; and in this chapter is narrated the case of an infant, infected by a syphilitic nurse-maid who kissed it, the result being that the child had a sore mouth! To the syphilitic affections of the eye and its appendages, other than iritis, but half of one page is devoted. The subject of nervous syphilis possesses no attractions for the author.

His reference to surgical authorities, whose names have long been unfamiliar to the average practitioner, is highly suggestive of the shelves of the dealer in second-hand books—the musty and calf-bound relics of the libraries of our fathers in medicine.

As for the illustrations, they seem to have been painted by an artist with few colors on his palette. The eruption represented on the integument of the man labelled "syphilitic lichen," appears to have been produced by the "running" of the vermilion which was used to color his cap; and the same inflammatory hue is depicted in the plates which are said to represent tubercular syphilis. The others are equally poor.

The truth is, that a correct representation of cutaneous diseases in color, is only obtainable by the expenditure of much money and more skill. The want of excellence in the plates before us is largely, though not exclusively, due to the fact that they were passed over too few stones. Students of cutaneous disease in this country have lately had an opportunity of observing good effects in the excellent plates of Dr. L. A. Duhring, of Philadelphia. And but few are aware of the labor expended by that gentleman in order to perfect his illustrations. After spending hours with the artist, he is often obliged to take the brush in his own hand, in order to give the finishing touch to the picture; and his lithographer has a *carte blanche* order to employ as many stones as are necessary to correctly reproduce the original drawing. For this purpose from ten to fifteen are often required (but two or three have been used in Dr. Durkee's illustrations), and the author feels obliged to superintend in person the printing of each impression from the stones.

We have read with interest the criticisms of this volume in its present edition, as they have thus far appeared in our current medical literature, and have failed to discover in any one a decision as to the real merits of the treatise.

We should recommend no one to procure it for his library who can purchase or obtain access to the other valuable works on the same subject in the English language, and cannot fail to express our surprise that the well-known publishers should have ventured to present this edition to the profession without having first insisted upon a most careful re-editing of its pages.

